# Poultry’s Persistence Problem: Drug-Resistant *Campylobacter* in Chicken

**Published:** 2005-05

**Authors:** Charles W. Schmidt

Mounting evidence suggests that the poultry industry’s use of antibiotics promotes antibiotic resistance among the foodborne bacteria that infect humans. One such bacterium is *Campylobacter*, a pathogen common to chicken products. Every year more than 1 million Americans develop *Campylobacter*-induced food poisoning from eating undercooked contaminated chicken. Resistant strains of *Campylobacter* are a growing public health threat, particularly among elderly and immunocompromised patients. This month, researchers from the Johns Hopkins Bloomberg School of Public Health provide evidence suggesting that chickens raised without antibiotics are less likely to carry antibiotic-resistant strains of *Campylobacter* **[*EHP*** **113:557–560]**.

The study focused on fluoroquinolones (FQs), a class of antimicrobials used to control the bacterium *Escherichia coli* in broiler chickens. Of the two FQs initially approved for use in poultry, Sara Flox WSP and Baytril, only the latter remains on the market. The Food and Drug Administration is seeking to repeal approval for Baytril due to concerns that it contributes to microbial resistance.

The authors collected chicken products from two “antibiotic-free” producers (Bell & Evans and Eberly Poultry) and two of the nation’s largest conventional producers (Tyson Foods and Perdue Farms). The conventional producers claimed to have stopped using FQs in February 2002. The authors began sampling chicken products one year later, in 2003. All samples were obtained from grocery stores in or near Baltimore, Maryland.

Chicken samples were processed using standard isolation techniques; however, at the final step, *Campylobacter* enrichments were streaked onto agar plates both with and without ciprofloxacin (a second-generation FQ used to treat human disease). The ciprofloxacin supplement enabled the authors to identify FQ-resistant *Campylobacter* isolates from among a mix of susceptible and resistant strains.

*Campylobacter* was detected on 84% of all the samples tested. FQ-resistant strains were detected on 17% using unsupplemented agar and on 40% using supplemented agar. Abstention from FQ use by poultry producers did not increase the likelihood of *Campylobacter* contamination. Moreover, conventional products were up to 460 times more likely to carry resistant strains than their antibiotic-free counterparts. Of particular interest is that FQ resistance in conventional products persisted for one year after cessation of industrial use.

Based on these findings, the authors suggest that even without antibiotics, resistant populations may remain prevalent over time. Persistence of these resistant populations may result from residual contamination in poultry houses, the authors suggest. For example, biofilms in water distribution systems can harbor *Campylobacter* and thus could serve as reservoirs for resistant populations. These findings suggest the need to further improve poultry house cleaning and disinfection, they write.

The authors say it is important to measure the prevalence of, and causes for, FQ-resistant strains in the food supply. To this end, they point out that supplemented agar may provide a much more sensitive tool than conventional methods for detecting resistant strains of the bacterium.

## Figures and Tables

**Figure:**
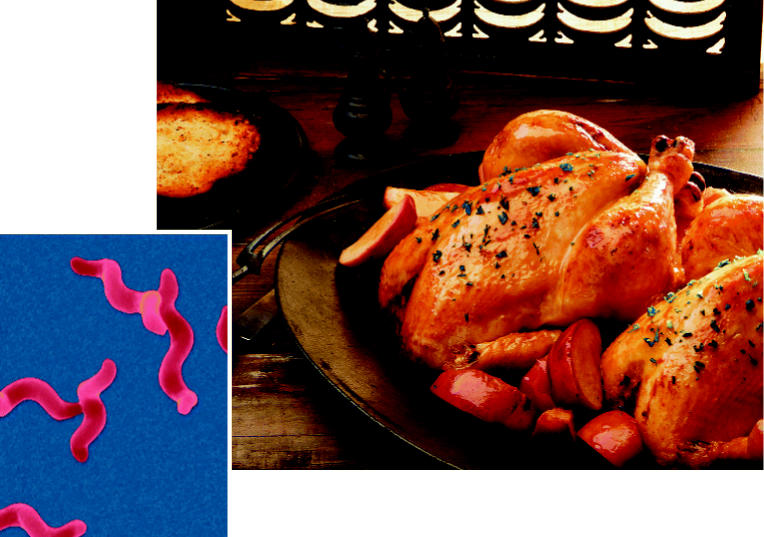
**Chicken surprise.** New data show that antibiotic-resistant *Campylobacter* (left) can persist in poultry populations—and products—long after producers stop using the drugs.

